# Changes in the mental health status of the general Chinese population during the COVID-19 pandemic: A longitudinal study

**DOI:** 10.3389/fpsyt.2022.765125

**Published:** 2022-07-28

**Authors:** Kun Jin, Jing Huang, Ziwei Teng, Fangtai Liu, Sujuan Li, Yan Qiu, Haishan Wu, Jindong Chen, Hui Xiang, Min Yang, Xuelei Xu, Hui Tang, Fangliu Shi

**Affiliations:** ^1^Department of Psychiatry, National Clinical Research Center for Mental Disorders, The Second Xiangya Hospital of Central South University, Changsha, China; ^2^Hunan Post and Telecommunication College, Changsha, China; ^3^Xiangshan Hospital of Traditional Chinese Medicine Medical Health Group (The Third Hospital District), Xiangshan, China

**Keywords:** COVID-19, mental health, general population, post-traumatic stress disorder, Chinese

## Abstract

The study is based on a longitudinal evaluation of the public, during the initial COVID-19 outbreak in China and 8 months after. It aimed to explore the changes in the mental health of the public at the beginning of the pandemic and during the regular epidemic prevention and control. An online survey questionnaire was used to collect data during the initial COVID-19 outbreak (February 10, 2020–February 18, 2020; T1) and 8 months after the outbreak (October 21, 2020–December 29, 2020; T2). Psychological distress was assessed using the Patient Health Questionnaire-9 (PHQ-9), Self-rating Anxiety Scale (SAS), and Post-traumatic Stress Disorder Checklist (PCL-5). A chi-square test was used to compare the changes in the depression and anxiety scores at T1 and T2, and the correlation between symptoms was analyzed through Spearman's rank correlation. In T1, 1,200 people were recruited, while 168 people responded in T2. Depression (48.2–31.0%; p=0.001) and anxiety (17.9–9.5%; *p* = 0.026) symptoms decreased over time; two participants developed post-traumatic stress disorder (PTSD) in T2. The scores of the PHQ-9 scale and the SAS scale were both positively correlated with the score of the PCL-5 scale and negatively correlated with sleep time. During the COVID-19 pandemic, part of the general population's anxiety and depression significantly reduced with time, and they rarely developed PTSD. PTSD occurrence was related to severe depression and anxiety.

## Introduction

In December 2019, a new type of coronavirus disease (COVID-19) was first detected in China, and it spread rapidly to more than 200 countries worldwide in the following 6 months. The COVID-19 pandemic has severely affected people's mental health. A meta-analysis involving 9,074 participants from 10 different countries demonstrated that during the COVID-19 pandemic, the prevalence of depression and anxiety was 33.7 and 31.9%, respectively (1). The main mental traumas that manifested in the general population were found to be emotional disturbance, depression, stress, low mood, irritability, insomnia, post-traumatic stress symptoms, anger, and emotional exhaustion, among which low mood and irritability were commonly reported (2).

While research on the mental health of the masses mainly comprises cross-sectional studies (3, 4), relatively few studies highlight how mental health has evolved during the COVID-19 pandemic. Although existing longitudinal studies have shown that psychological problems increased during the early stages of the pandemic, it is still unclear how individuals' mental health changed a few months after the outbreak (5–7). A longitudinal survey of 17,761 adults in the United Kingdom showed that at the beginning of the lockdown in April 2020, more than one-third of participants had mental health problems, including depression and anxiety symptoms. However, with the lifting of the blockade restrictions in May 2020, the psychological problems gradually decreased. By July 2020, only a quarter of the participants were found to have psychological problems (8). Another 12-week study conducted in Australia showed similar results. Based on a psychological evaluation of 5,455 participants, anxiety symptoms increased slightly, but significantly, in the first 4 weeks after the COVID-19 outbreak, and they gradually decreased to the baseline level in the following weeks (9). However, Gopal et al. (10) found that symptoms of stress, depression, and anxiety significantly increased 2 months after the outbreak. The author believes that this may be related to the nationwide lockdown and the sharp increase in the number of COVID-19 cases. In general, with the spread of COVID-19, there is still no unified conclusion regarding what direction the psychological conditions are taking. Therefore, more widescale longitudinal studies on the matter are needed urgently.

In China, the government initiated the first-level response to major public health emergencies in many areas across the country, from January 23, 2020, to May 2, 2020, and it adopted restrictive policies such as restrictions on movement, social distancing, and quarantining of contacts, in response to the COVID-19 outbreak. These multiple pressures negatively affected the behaviors of individuals and caused psychological problems (e.g., panic, anxiety, helplessness, and irritability) (11). Studies have found that in the early stages of the epidemic, the general population in China suffered from moderate to high levels of psychological distress. However, with time, fear gradually decreased, but the level of depression increased significantly (12). Nonetheless, most studies only focus on the changes in mental health during the early stages of the COVID-19 pandemic (13–15), and relevant research on the long-term changes in these psychological symptoms is scarce.

This study aimed to monitor the changes in the mental health of the Chinese public through a longitudinal evaluation at the onset of the COVID-19 pandemic in China and 8 months after the outbreak. The current study hypothesized that the mental health of the general population in China improved over time, compared to the period at the beginning of the lockdown.

## Methodology

### Study settings and participants

This longitudinal study conducted an online questionnaire over two periods—during the early stage of the COVID-19 pandemic (February 10, 2020–February 18, 2020; T1) and 8 months after the outbreak (October 21, 2020–December 29, 2020; T2). Questionnaires were uploaded to a popular online professional survey platform “Wenjuanxing” (www.wjx.cn) and distributed to the general Chinese population *via* e-mail or WeChat. The questionnaire comprised 28 questions in four categories, including sociodemographic information, behavior response, public perceptions in response to the COVID-19 vaccine, and the mental health status of the participants. The participants were informed of the purpose of the study, and they provided informed consent before the investigation began. The inclusion criteria were: (1) age range between 18 and 60 years; (2) can understand Chinese and use a smartphone; (3) willing to participate and have signed the electronic informed consent. The exclusion criterion was cognitive impairment hindering comprehension of the questionnaire. All questionnaires were filled out anonymously and were kept strictly confidential. While 1,200 individuals answered the questionnaire for T1, only 607 participants provided their contact information and were willing to participate in the follow-up survey. For T2, 168 individuals participated in the survey. The study was approved by the ethics committee of the Second Xiangya Hospital of Central South University.

### Measures

#### Sociodemographic information

The first part of the online questionnaire included questions related to sociodemographic information, including gender, age, marital status, education level, household registration, occupation, and infection status.

#### Behavior response

This part of the self-reported questionnaire comprised six statements distributed over two response domains: Sleep performance and concerns about COVID-19. For sleep performance, participants were asked how satisfied they were with their sleep over the past 6 months, how long they slept, and whether they used medication to help them sleep. Regarding concerns about COVID-19, participants were asked how concerned they were about COVID-19, how worried they were about a resurgence of the epidemic, and how prepared they were to prevent a resurgence. Specific questions and options are shown in **Table 3**.

#### Public perceptions in response to the COVID-19 vaccine

This part includes the following three questions: (1) How concerned were they about the COVID-19 vaccine? (2) How necessary was it to develop the COVID-19 vaccine? (3) How necessary was it to be vaccinated? The items were scored based on a five-point scale, comprising “strongly disagree, tend to disagree, neither agree nor disagree, tend to agree, and strongly agree.”

#### Depressive symptoms (patient health questionnaire-9)

Depressive symptoms were assessed using the Patient Health Questionnaire-9 (PHQ-9) scale. It contains nine items based on the diagnostic criteria for Major Depressive Disorder in the Diagnostic and Statistical Manual of Mental Disorders (DSM-IV) developed by the American Psychiatric Association. Items are scored from 0 to 3, and the highest total score is 27 points. The scores were categorized as follows: mild depression (5–9 points), moderate depression (10–14 points), and severe depression (15–27 points). The Chinese version of the PHQ9 scale has good construct validity, and its Cronbach's α coefficient was 0.892 (16). In this study, a 5-point cut-off score was used to distinguish between participants with normal emotions and those with depressed emotions.

#### Anxiety (self-rating anxiety scale)

Anxiety symptoms were assessed using the Self-rating Anxiety Scale (SAS). The scale comprises 20 items scored from 1 to 4 points. The total score is then multiplied by 1.25, and the integer is used to calculate the standard score. The criterion validity of the Chinese version of the SAS scale compared with the Hamilton Anxiety Rating Scale (HAM-A) was 0.365, indicating high validity, and the Cronbach's α coefficient was 0.862 (17). According to the results of the Chinese norm, the threshold of the SAS standard score is 50 points. The scores were categorized as follows: mild anxiety (50–59 points), moderate anxiety (60–69 points), and severe anxiety (70 points and above). In this study, a 50-point cut-off score was used to indicate whether participants had anxiety.

#### Post-traumatic stress disorder (post-traumatic stress disorder checklist)

Post-traumatic stress disorder (PTSD) refers to a stress-related disorder that occurs after an individual experiences abnormally strong mental stimulation, and it is assessed using the self-rated Post-traumatic Stress Disorder Checklist (PCL-5) (18). It includes four dimensions: intrusive symptoms, avoidance symptoms, symptoms of negative changes in cognition and emotion, and symptoms of excessive arousal. The PCL-5 comprises 20 items that are scored from 0 to 4 points. The Chinese version of the PCL-5 scale has good convergent validity and discriminant validity, and its Cronbach's α coefficient was 0.91 (19). If the total score was ≥33 points, they were classified as having PTSD, with higher scores indicating more severe symptoms.

### Statistical analysis

All statistical analyses were performed using SPSS version 23 (IBM Corp, Armonk, NY). Continuous variables were shown as means and standard deviations, while categorical variables were shown as numbers and percentages. The chi-square test and Wilcoxon rank-sum test were used to compare the changes in depression and anxiety scores in T1 and T2. The Spearman's rank correlation was used to analyze the correlation between depression, anxiety, PTSD, behavior response, and public perceptions. Finally, a logistic regression (unadjusted and adjusted) was performed to estimate the odds ratio of risk factors and their impact on depression and anxiety.

## Results

The participants' characteristics are shown in Table 1. The average age of the participants was 28.1, of which 81.5% had a college degree or higher. The ratio of male to female participants was roughly equal.

**Table 1:** Baseline characteristics of the 168 study participants.

**Variables**	
Gender
Male	86 (50.2)
Female	82 (48.8)
Age (years)	28.05 ± 8.43
**Residence**
Hubei province	75 (44.6)
Others	93 (55.4)
Education
**Below university**	31 (18.5)
College	99 (58.9)
Master's or doctorate	38 (22.6)
**Marital status**
Unmarried	110 (65.5)
Married	56 (33.3)
Others^a^	2 (1.2)

a *Means divorced, widowed*.

Table 2 shows that T1 participants' average PHQ-9 score was 5.83 (SD = 5.95), and 48.2% of these participants had depressive symptoms. T2 participants' average PHQ-9 score was 3.23 (SD = 4.12), and 31.0% of the participants had depressive symptoms. T1 participants' average SAS score was 41.35 (SD = 10.97), and 17.9% of them had anxiety symptoms. T2 participants' average SAS score was 40.35 (SD = 8.45), and 9.5% of the participants had anxiety symptoms. The chi-square test results indicated statistical differences between the depression score and anxiety score (pPHQ-9 = 0.001; pSAS = 0.026) in T1 and T2, indicating that the participants' depression and anxiety levels decreased over time. Wilcoxon rank-sum test showed that the depression score in T2 was significantly lower than that in T1 (Z = −5.467; *p* < 0.001). T2 participants' average score of PCL-5 was 5.32 (SD = 7.02), and only two participants scored more than 33 points.

**Table 2:** Depression, anxiety, and PTSD in study participants.

**Variables**	**COVID-19 outbreak**		**COVID-19 outbreak after 8 months**		χ^2^	**Z**
	**Mean**	**Number (%)**	**Mean**	**Number (%)**		
Depression	5.83 ± 5.95	81 (48.2)	3.23 ± 4.12	52 (31.0)	10.466^**^	−5.467^***^
Mild		39 (23.2)		41 (24.4)		
Moderate		26 (15.5)		6 (3.6)		
Severe		16 (9.5)		5 (3.0)		
Anxiety	41.35 ± 10.97	30 (17.9)	40.35 ± 8.45	16 (9.5)	4.937^*^	−0.336
Mild		19 (11.3)		12 (7.1)		
Moderate		8 (4.8)		2 (1.2)		
Severe		3 (1.8)		2 (1.2)		
PTSD (PCL5)		5.32 ± 7.02	2 (0.1)		

*Z: Wilcoxon rank-sum test*.

*** *p <0.001*.

** *p <0.01*.

* *p <0.05*.

Table 3 shows the behavior response to the COVID-19 outbreak after 8 months. Of the participants, 10.7% reported poor sleep during the last 6 months, and 6% used drugs to help them sleep. In addition, 76.8% of the participants were still worried about the COVID-19 outbreak 8 months later, and 88.1% reported preparing in advance for another possible outbreak.

**Table 3:** Behavior response to COVID-19 outbreak after 8 months.

**Variables**	**Options (number, percentage)**				
Have you ever infected with COVID-19?	Confirmed infection (0)	Close contact with COVID-19 patients (1, 0.6)	Suspected infected person (0)	Frontline staff (5, 3.0)	No infection (162, 96.4)
How about your sleep in the last 6 months?	Very good (39, 23.2)	Commonly (111, 66.1)	Poor (17, 10.1)	Very poor (1, 0.6)	
How much sleep do you keep every day in the last 6 months?	<4 h (1, 0.6)	4–6 h (26, 15.5)	6–8 h (121, 72)	>8 h (19, 11.3)	Uncertain (1, 0.6)
Have you ever used drugs to help you sleep?	Frequently used (1, 0.6)	Occasionally used (9, 5.4)	Never (158, 94)		
Do you pay close attention to COVID-19 around the world?	Very concerned (28, 16.6)	Concerned (30, 17.9)	Occasional attention (80, 47.6)	No attention (30, 17.9)	
Are you worried that COVID-19 will explode again?	Very worried (22, 13.1)	A little worried (107, 63.7)	Don't worried (20,11.9)	worried about other similar outbreaks (19, 11.3)	
Are you prepared for a possible outbreak of COVID-19?	Buy masks (117, 69.6)	Buy the necessary medicine (62, 36.9)	Buy enough living materials (50, 29.8)	Wait until the COVID-19 outbreak (42, 25)	not prepared (20, 11.9)

Table 4 shows that 63.7% of the participants often paid attention to information about the COVID-19 vaccine, 89.3% felt that the development of the COVID-19 vaccine was necessary, and 67.4% were eager to get vaccinated.

**Table 4:** Public perceptions in response to the COVID-19 vaccine.

	**Strongly disagree**	**Tend to disagree**	**Neither agree nor disagree**	**Tend to agree**	**Strongly agree**
Pay attention to the information about the COVID-19 vaccine	12 (7.1)	18 (10.7)	31 (18.5)	76 (45.2)	31 (18.5)
Do you think the research and development of the COVID-19 vaccine is very necessary?	6 (3.6)	3 (1.8)	9 (5.3)	43 (25.6)	107 (63.7)
Do you think it is necessary for you to be vaccinated?	6 (3.6)	5 (3.0)	44 (26.0)	51 (30.4)	62(37.0)

Table 5 shows the correlations of demographic characteristics with depression and anxiety. Participants' scores on the PHQ-9 scale were positively correlated with the scores on the PCL-5 scale (*r* = 0.685, *p* < 0.01), and negatively correlated with sleep time (*r* = −0.239, *p* < 0.01). The SAS scores were positively correlated with the PCL-5 scores (*r* = 0.595, *p* < 0.01), and negatively correlated with sleep time (*r* = −0.186, *p* < 0.05). The SAS scores were positively correlated with the degree of attention to the epidemic (*r* = 0.187, *p* < 0.05); that is, the more concerned people are about the pandemic, the more anxious they are.

**Table 5:** Correlations of PTSD and behavior response with depression and anxiety.

**Factors**	**PTSD (PCL5)**	**How much sleep do you keep every day in the last 6 months?**	**Do you pay close attention to COVID-19 around the world?**
Depression	0.685^**^	−0.239^**^	0.116
Anxiety	0.595^**^	−0.186^*^	0.187^*^

** *p <0.01*.

* *p <0.05*.

Table 6 shows the effects of behavior response on depression and anxiety. After adjusting for demographic characteristics such as gender, age, education, residence and marital status, sufficient sleep duration was significantly associated with a lower risk of depression [OR (95% CI) = 0.352 (0.173–0.716)] and anxiety [0.258 (0.083–0.803)]. Excessive focus on the epidemic was significantly associated with increased risk of anxiety [2.089 (1.226–3.560)].

**Table 6:** Effects of behavior response on depression and anxiety.

	**How much sleep do you keep every day in the last 6 months?**	**Do you pay close attention to COVID-19 around** **the world?**
**Depression**		
Crude OR [95% CI]	0.396 [0.208–0.756]	–
Adjusted OR [95% CI]	0.352 [0.173–0.716]	–
**Anxiety**		
Crude OR [95% CI]	0.382 [0.148–0.990]	1.878 [1.165–3.025]
Adjusted OR [95% CI]	0.258 [0.083–0.803]	2.089 [1.226–3.560]

## Discussion

This study compared the psychological conditions of the Chinese general population in the early stage of the COVID-19 pandemic and 8 months after the outbreak. The participants' mental health was found to improve significantly in the phase of regular epidemic prevention and control. During the 8 month study period, the number of participants with depression and anxiety decreased significantly, and most of them did not develop PTSD after the outbreak, which is consistent with our initial assumption. The study also found that the improvement in depression and anxiety symptoms was positively correlated with the reduction in PTSD symptoms and negatively correlated with the length of sleep. The increase in anxiety is also positively correlated with the degree of attention to the pandemic. At the same time, most of the participants maintained continuous attention to the progress of the pandemic and the development of vaccines.

People may suffer from various mental health problems after a natural disaster or a pandemic due to infectious diseases (20–22). A longitudinal study showed that the anxiety level of ordinary residents in Hong Kong increased significantly when the severe acute respiratory syndrome (SARS) epidemic first broke out, but then gradually decreased over time (23). Our study's findings are in line with these results. A possible reason for this is that a more in-depth understanding of the pandemic may have alleviated the fear of the unknown (24). Moreover, lifting the restrictive measures allowed people to resume work and ordinary life, and is most likely a contributory factor as well. A normal sleep schedule, social interaction, exercise, and reduced financial burden have all contributed to the collective improvement of the population's mental health (25, 26). However, a study conducted in Austria around the same time showed different results. The study evaluated the psychological conditions of 437 Austrians after 4 weeks (April 10, 2020) and 6 months (September 7, 2020) of lockdown in the local area. No statistical difference was found between participants with symptoms of depression, anxiety, and insomnia at the two time points. Six months after the outbreak, mental health problems had remained at a high level (27). This may be because the effective results from the Chinese government's steady advancement of preventive measures, including isolating the source of infection; increasing subsidized medical treatment expenses for COVID-19 patients; and releasing epidemic information in a timely, open, and transparent manner have provided positive psychological support to the public. Another possible reason is that our research has different time points; our study assessed the mental health of the general population around November 2020. At this time, vaccines against COVID-19 had been developed and preliminary results had been obtained, which effectively reduced fear and anxiety regarding COVID-19.

Numerous studies have shown that exposure to severe trauma often leads to PTSD. A review has shown that the general population had the lowest PTSD incidents after a disaster, compared to direct victims or rescuers, which is between 5 and 10%, respectively (20). As previously mentioned, among the 168 participants in this study, only two developed PTSD. At the beginning of the outbreak, more than 20% of the participants had moderate to high levels of depression and anxiety, while at the second follow-up, the number dropped to below 10%. Therefore, the relief of anxiety and depression may reduce the occurrence of PTSD in individuals. Previous studies support this conclusion. For instance, Breslau et al. (28) conducted a 5-year follow-up survey of 107 adults and found that people with severe depression have a higher risk of PTSD than people without depression. Schindell-Allon et al. (29) proposed a “depressogenic model,” which indicated a causal relationship between depression and PTSD; that is, initial major depression can lead to subsequent PTSD. Hence, positive emotions can contribute to post-traumatic recovery.

Additionally, we found that the improvement of depression and anxiety symptoms was negatively correlated with the length of sleep. Changes in sleep are related to the occurrence and development of many diseases (30–33), especially in affect-related disorders. In fact, depression and anxiety are often intertwined with sleep problems (34, 35). Poor sleep quality, insomnia, and extended or insufficient sleep are all risk factors for depressive episodes (36–38). Trabelsi et al. (39) found that sleep quality could predict changes in the mental health of the elderly during the recent coronary pneumonia epidemic. Additionally, a survey of 751 pregnant women in Shenzhen during the COVID-19 pandemic showed that difficulty falling asleep, short sleep duration, and poor subjective sleep quality were all closely related to anxiety and depressive symptoms (40). In our study, 83.9% of the participants slept for more than 6 h. Having sufficient sleep duration may help explain the improved mental health of these participants.

This study also investigated the participants' views on the COVID-19 vaccine and found that most participants perceived vaccine development as necessary, and they wished to receive the vaccine. The development of vaccines not only directly reduces the psychological pressure of the pandemic on the public, but also plays an indirect role in supporting the public's mental health. As such, public awareness of the importance and role of vaccines contributes to the prevention and control of the COVID-19 pandemic.

This study had some limitations. First, the sample size is quite small, and the response rate of the second survey was relatively low (about 27.6%). This may be because, for the second survey, we sent the questionnaires *via* email; thus, mostly younger individuals with a college education or above responded because, compared to other groups, they use emails more often and are more likely to accept online surveys. Second, this study only discussed the changes in the mental health of the general population during the COVID-19 pandemic; thus, the mental health status of the participants at the baseline (i.e., before the pandemic) was not studied. Third, this study considered depression, anxiety, PTSD, and other scores overall evaluate the participants' mental health. However, there is some duplicate content between the subscales of these three scales. In the future, we recommend analyzing the relationship between symptoms in more detail from each dimension in the scale. Moreover, standardized symptom assessment tools, such as the Epworth Sleepiness Scale, Insomnia Severity Index, or Pittsburgh Sleep Quality Index are useful in providing an objective appraisal of sleep symptoms.

This study tracked the psychological changes of ordinary Chinese people during the outbreak and remission of COVID-19. The study found that during the COVID-19 pandemic, anxiety, and depression among part of the general population significantly reduced with time, and PTSD rarely occurred. At present, global prevention measures are still in effect, and people are worried about another outbreak. In the future, the public will be in a long-term normalized state of active epidemic prevention. Our research provides a reference for understanding the changes in the general population's mental health during the pandemic, which is conducive to better public health interventions.

## Data availability statement

The original contributions presented in the study are included in the article/supplementary material, further inquiries can be directed to the corresponding authors.

## Author contributions

JH and ZT contributed to conception and design of the study. KJ and ZT contributed to data collection and statistical analysis. SL, YQ, HW, HX, MY, and XX contributed to the literature review. KJ contributed to writing of the manuscript. JH, FL and HT contributed to revising the manuscript. JC, HT, and FS provided funds to conduct the study. All authors have approved the final version of the manuscript.

## Funding

The study was supported by the National Nature Science Foundation of China (Grant Nos. 81901401, 81971258, and 82101578).

## Conflict of interest

The authors declare that the research was conducted in the absence of any commercial or financial relationships that could be construed as a potential conflict of interest.

## Publisher's note

All claims expressed in this article are solely those of the authors and do not necessarily represent those of their affiliated organizations, or those of the publisher, the editors and the reviewers. Any product that may be evaluated in this article, or claim that may be made by its manufacturer, is not guaranteed or endorsed by the publisher.
